# Increased Epithelial Expression of CTGF and S100A7 with Elevated Subepithelial Expression of IL-1β in Trachomatous Trichiasis

**DOI:** 10.1371/journal.pntd.0004752

**Published:** 2016-06-01

**Authors:** Tamsyn Derrick, Philip J. Luthert, Hodan Jama, Victor H. Hu, Patrick Massae, David Essex, Martin J. Holland, Matthew J. Burton

**Affiliations:** 1 Department of Clinical Research, Faculty of Infectious and Tropical Diseases, London School of Hygiene and Tropical Medicine, London, United Kingdom; 2 Kilimanjaro Christian Medical Centre, Moshi, Tanzania; 3 UCL Institute of Ophthalmology, London, United Kingdom; 4 International Centre for Eye Health, Department of Clinical Research, Faculty of Infectious and Tropical Diseases, London School of Hygiene and Tropical Medicine, London, United Kingdom; University of California San Diego School of Medicine, UNITED STATES

## Abstract

**Purpose:**

To characterize the histological appearance and expression of pro-inflammatory mediators, growth factors, matrix metalloproteinases and biomarkers of epithelial-mesenchymal transition (EMT) in healthy control and trachomatous trichiasis (TT) conjunctival tissue.

**Methods:**

Conjunctival biopsies were taken from 20 individuals with TT and from 16 individuals with healthy conjunctiva, which served as controls. Study participants were of varying ethnicity and were living in a trachoma-endemic region of northern Tanzania. Formalin-fixed paraffin-embedded tissue sections were stained using hematoxylin and eosin or by immunohistochemistry using antibodies against: IL-1β, IL-6, IL-17A, IL-22, CXCL5, S100A7, cleaved caspase 1 (CC1), PDGF, CTGF, TGFβ2, MMP7, MMP9, E-cadherin, vimentin, and αSMA.

**Results:**

Tissue from TT cases had a greater inflammatory cell infiltrate relative to controls and greater disruption of collagen structure. CTGF and S100A7 were more highly expressed in the epithelium and IL-1β was more highly expressed in the substantia propria of TT cases relative to controls. Latent TGFβ2 was slightly more abundant in the substantia propria of control tissue. No differences were detected between TT cases and controls in the degree of epithelial atrophy, the number of myofibroblasts or expression of EMT biomarkers.

**Conclusions:**

These data indicate that the innate immune system is active in the immunopathology of trachoma, even in the absence of clinical inflammation. CTGF might provide a direct link between inflammation and fibrosis and could be a suitable target for therapeutic treatment to halt the progression of trachomatous scarring.

## Author Summary

Progressive scarring of the conjunctiva in individuals with trachoma causes the eyelids to contract, drawing the eyelashes inwards (trichiasis) so that they scratch the cornea, causing pain and eventually blindness. Disease is initiated in childhood by repeated conjunctival infection with *Chlamydia trachomatis* (Ct), however, infection is not commonly found in adults, yet chronic inflammation and fibrosis progress throughout the lives of many individuals. A better understanding of the mechanisms driving inflammation and fibrosis are required in order to develop treatments to halt disease progression. The tissue expression and localization of a number of pro-inflammatory cytokines, growth and matrix factors were investigated in eyelid tissue from 20 individuals with trichiasis and from 16 control individuals. By staining tissue sections with dyes and specific antibodies, pro-inflammatory signaling molecules IL-1β and S100A7 and pro-fibrotic growth factor CTGF were found to be more highly expressed in individuals with trichiasis. CTGF and S100A7 were highly expressed in the epithelium; the outermost layer of the conjunctiva, whereas IL-1β was more highly expressed deeper in the tissue, where scarring occurs. Numerous inflammatory cells were found in the tissue of trichiasis patients even in the absence of clinically apparent inflammation. Future research should seek to describe a causative mechanism linking these factors.

## Introduction

Trachoma is a blinding disease initiated by infection of the conjunctival epithelium with the intracellular bacterium *Chlamydia trachomatis* (Ct). Individuals living in trachoma-endemic communities are repeatedly infected with Ct, which causes a follicular conjunctivitis. Chronic, recurrent inflammation, even in the absence of detectable Ct infection, is associated with progressive scarring [1]. The fibrotic response results in the inward turning of the lid margin (entropion) and abrasion of the cornea by the eyelashes (trichiasis). Mechanical damage to the cornea and subsequent opportunistic infections eventually lead to corneal opacity and blindness.

Trachoma is endemic in 51 countries and impairs the eyesight of 2.2 million people worldwide, 1.2 million of whom are irreversibly blind [2]. Although trachoma control programs have made good progress in reducing active disease, there is now some evidence that established scarring disease continues to progress even when chlamydial infection appears well controlled [1]. Therefore, a large number of people remain at risk of developing incident trichiasis, especially in areas where mass drug administration has had a partial effect [3,4]. In order to develop a vaccine or therapeutic treatments to prevent the progression to trichiasis, a better understanding of the immunopathology of scarring trachoma is required.

A number of clinical studies have shown that transcriptional signatures in trachomatous scarring (TS) and trichiasis (TT) are consistent with a pro-inflammatory epithelial response and tissue remodeling, supporting the cellular paradigm of chlamydial disease pathogenesis [5]. The gene expression of a number of pro-inflammatory mediators (*IL17A*, *IL1B*, *CXCL5*, *S100A7* (psoriasin), growth factors (*CTGF* (connective tissue growth factor)) and matrix metalloproteinases (*MMP7*, *MMP9*) were up-regulated in TS and TT [1,6–9]. Expression was increased further when clinical inflammation was present [1,6,7]. Immunohistochemistry (IHC) studies using tissue from a small number of individuals with active trachoma have shown that MMP9, CTGF, platelet derived growth factor (PDGF) and IL-1β were up-regulated in infiltrating monocytes/macrophages and that IL-1β was increased in the conjunctival epithelium [10–12].

Inflammatory mediators, growth factors and MMPs can stimulate epithelial cells to differentiate into pro-fibrotic mesenchymal cells, a process known as epithelial-mesenchymal transition (EMT) [13–15]. Epithelial cells undergoing EMT lose expression of E-cadherin and gain mesenchymal ‘expression’ markers vimentin and α-smooth muscle actin (αSMA) as they migrate through the basement membrane into the stroma, where they contribute to fibrosis [16]. Inflammation-induced EMT normally ceases when inflammation resolves; therefore EMT only becomes pathological in an environment of chronic inflammation. The evidence of chronic pro-inflammatory cytokine and growth factor expression in various stages of trachoma combined with a fibrotic tissue response suggests that EMT may contribute to the pathology of trachoma.

The aim of this IHC study of trachomatous conjunctival tissue was to investigate the relative protein level and tissue localization of pro-inflammatory mediators, growth factors, EMT biomarkers and MMPs and to characterize the changes in tissue architecture that occur in TT. Molecular markers studied include factors that were previously shown to be up-regulated in TS/TT (S100A7, IL-1β, IL-17A, CXCL5, CTGF, MMP7/9), EMT biomarkers (αSMA, vimentin, E-cadherin) and other factors that may play a role in immunopathology (IL-6 (pleiotropic pro-inflammatory cytokine), IL-22 (mucosal defense and epithelial integrity), PDGF, transforming growth factor beta 2 (TGFβ2) (both growth factors associated with fibrosis), and cleaved Caspase 1 (CC1), a marker of inflammasome activation).

## Methods

### Ethical permission

This study adhered to the tenets of the Declaration of Helsinki and was approved by the London School of Hygiene and Tropical Medicine Ethics Committee, the Tanzanian National Institute of Medical Research Ethics Committee and the Kilimanjaro Christian Medical Centre Ethics Committee. Written, informed consent was obtained from individuals before enrollment in the study.

### Clinical assessment and biopsy sampling

Study participants were examined using a bright torch and x2.5 loupes. The clinical phenotype of individuals for follicles, papillary inflammation and trichiasis was graded using the World Health Organization 1981 FPC trachoma grading system [17]. Conjunctival scarring was graded in finer detail using the system described by Hu *et al* [18]. Biopsy samples were collected from individuals undergoing bilamellar tarsal rotation surgery for TT (cases) and from individuals without clinical evidence of trachoma undergoing cataract surgery (controls), matched by age and sex where possible. The eyelid was anaesthetized with an injection of 2% lignocaine (Vital Healthcare, Mumbai, India) and the eye was cleaned with 5% povidone iodine. Biopsy samples were taken from the upper tarsal conjunctiva using a 3mm trephine: 2mm from the lid margin at the junction of the medial ⅔ and lateral ⅓ of the everted lid. Samples were fixed in 10% neutral buffered formalin and subsequently embedded in paraffin wax.

### Staining

Formalin-fixed paraffin-embedded (FFPE) samples were cut perpendicular to the conjunctival surface in 4μm thick sections. Sections were stained with hematoxylin and eosin (H&E) for examination of tissue health and composition. Sections for IHC staining were dewaxed and stained with antibodies for pro-inflammatory cytokines and chemokines (IL-6, IL-1β, IL-17A, IL-22, CXCL5), antimicrobial peptide psoriasin (S100A7), cleaved caspase 1 (CC1), growth factors (PDGF, CTGF, TGFβ2), matrix metalloproteinases (MMP7, MMP9) and biomarkers of EMT (E-cadherin, vimentin, αSMA). Antibodies and retrieval methods used are listed in S1 Table. IHC staining was automated and performed using Novocastra Bond Polymer Refine Red Detection reagents on a Leica BOND instrument (Leica Biosystems, Milton Keynes, UK). Sections were covered with a cover-slip for microscopic examination.

### H&E grading protocol

Tissue sections were graded by an ophthalmic pathologist masked to the clinical status of the samples. Where more than one H&E section was available for a sample the slide with the most tissue was analyzed. H&E slides were graded on a scale of 0 to 3 for the degree of epithelial atrophy (where 0 is none and 3 is severe atrophy), the number of inflammatory cells present and the number of myofibroblasts present (0 = no visible staining, 1 = few cells, 2 = moderate number of cells and 3 = abundant cells). H&E sections were viewed under cross-polarized light in order to view collagen fiber deposition and grade fibrosis. Fibrotic scarring was graded for 3 patterns, ‘block’, ‘wavy’ and ‘fine’, each on a scale of 0 to 3: 0 = none seen, 1 = focal patches, 2 = abundant areas and 3 = extensive.

### IHC grading protocol

Antibodies were graded according to strength and location of staining. For each antibody the section was graded separately for the epithelial and the subepithelial compartments. The subepithelial compartment (substantia propria) contained the stroma and inflammatory cell infiltrate if present. Antibody staining was recorded on a scale of: 0 = no visible staining, 1 = few cells, 2 = moderate number of cells and 3 = abundant cells. For the antibodies targeting E-Cadherin, vimentin and αSMA staining was recorded in the epithelial compartment only. For E-cadherin, the total area of the epithelium that stained positive was recorded in quartiles: 0–25% = 1, 26–50% = 2, 51–75% = 3 and 76–100% = 4.

### Data analysis

Data were analysed in R (https://www.r-project.org). Fishers Exact Tests were used to test for differences between case-control status and: age (categorized by decade), sex, ethnic group, H&E and IHC scores. IHC targets were excluded from the analysis where ≤2/36 sections had a grade >0. An unadjusted P value of <0.05 was considered statistically significant for hypothesis-generating purposes. Radial plots were generated by calculating the average score per person for TT cases and controls for each antibody or H&E feature.

## Results

### Sample phenotypes

Thirty-six conjunctival biopsy specimens were collected from 20 individuals with TT (cases) and 16 individuals with no clinical signs of trachoma (controls). The demographic and clinical phenotypes of individuals whose samples were used in this study are described in Table 1. There was no significant difference in sex (P = 0.31) or age (P = 0.074) between cases and controls. There was a significant difference in ethnic groups between cases and controls (p<0.0001); 18/20 cases were of the Massai ethnic group whereas only one control subject was Massai. No follicles were detected in cases or controls. One TT case had a papillary inflammation grade of 3, equivalent to *trachomatous inflammation–intense* using the simplified grading system [19]. All cases had varied degrees of conjunctival scarring. None of the controls had papillary inflammation, scarring or trichiasis (Table 1).

**Table 1:** Demographic and clinical characteristics of samples.

Variable		Cases	Controls
		N = 20	N = 16
**Gender, male (%)**		7 (35%)	9 (56%)
**Age, mean in years (range)**		74.6 (41–91)	70.3 (50–83)
**Ethnic group**			
	Massai	18	1
	Chagga	2	7
	Other	0	8
**Scarring grade**			
	0	0	16
	S1b	4	0
	S1c	8	0
	S2	3	0
	3	5	0
**Papillary Inflammation grade**			
	0	9	16
	1	8	0
	2	2	0
	3	1	0
**Trichiasis Grade**			
	0	2	16
	1	1	0
	2	9	0
	3	8	0

2 TT cases that scored 0 for trichiasis grade had marked entropion and had epilated lashes.

### Tissue morphology

**Fig 1:**
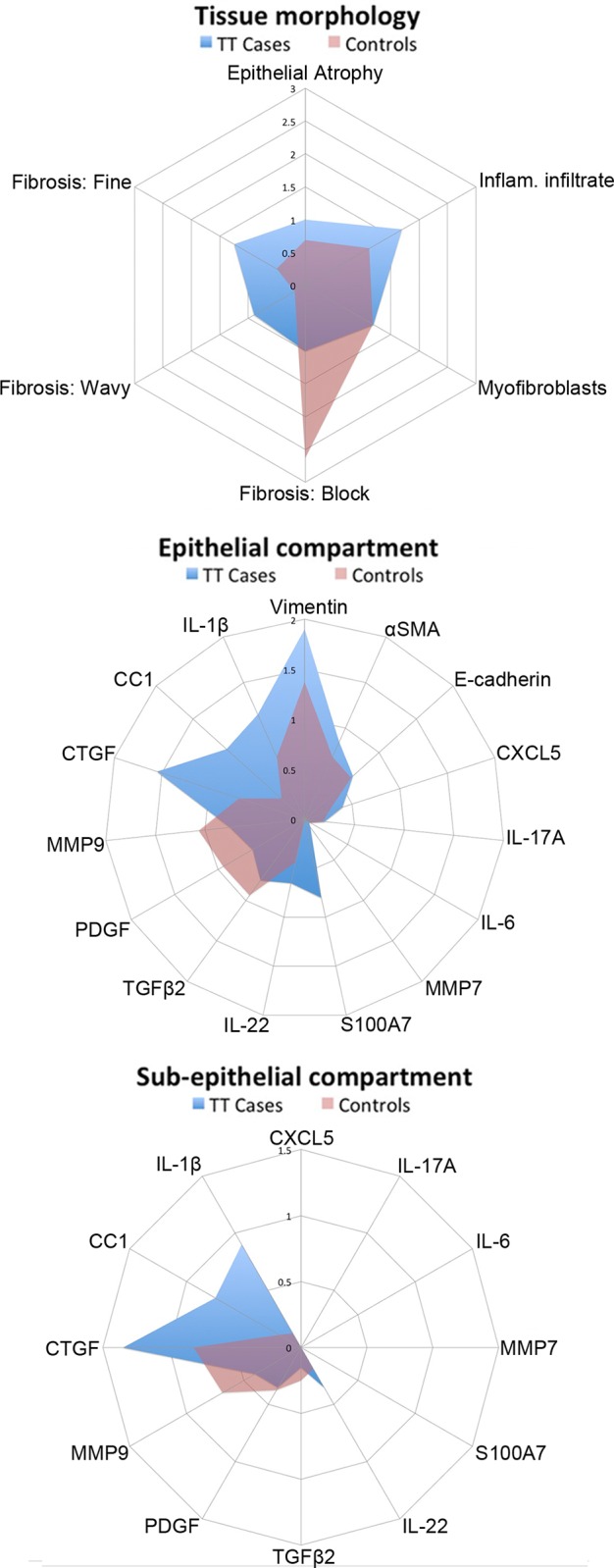
Radial plots summarizing overall changes in tissue morphology and expression of molecular markers. Molecular marker expression was quantified by IHC in the epithelial and subepithelial compartments. The average score per person for TT cases (blue) and controls (red) was plotted for each target or H&E feature.

H & E staining was used to visualize tissue structure and prevalence of inflammatory cells in sections. There was no difference in the degree of epithelial atrophy or in the number of myofibroblasts between cases and controls (Table 2). There were significantly more inflammatory cells evident in cases (P = 0.001). Three patterns of subepithelial tissue deposition became apparent when sections were viewed under cross-polarized light: “block”, “wavy” and “fine”. Representative photographs of these phenotypes are shown in S1 Fig. Tissue from cases had significantly more wavy (P = 0.0075) and fine patterns (P = 0.0005) of subepithelial tissue deposition, whereas individuals with healthy conjunctiva had more block type patterns (P = 0.0005), Table 2 and Fig 1.

**Table 2:** Sample characteristics by Hematoxylin and Eosin staining.

		Cases	Controls	P value^*^
**Epithelial Atrophy**				0.65
	0	6	7	
	1	7	3	
	2	5	4	
	3	1	0	
	NA^†^	1	2	
**Inflammatory cell infiltrate**				0.0025
	0	0	1	
	1	7	13	
	2	12	1	
	3	1	1	
**Myofibroblasts**				1
	0	2	2	
	1	8	7	
	2	8	6	
	NA^†^	2	1	
**Fibrosis: Block pattern**				0.0005
	0	5	0	
	1	11	1	
	2	3	4	
	3	1	11	
**Fibrosis: Wavy pattern**				0.0075
	0	6	13	
	1	10	3	
	2	4	0	
**Fibrosis: Fine pattern**				0.0005
	0	0	9	
	1	15	6	
	2	5	1	

^*^ Fishers exact test was used to test for differences between groups. Unadjusted P values are shown.

^†^ Sections received “NA” when there was not enough tissue present to grade a parameter and were not included in the significance calculation.

### Distribution and localization of molecular markers in conjunctival tissue

The relative expression of the different molecular markers detected by IHC in the epithelial and subepithelial compartments were analysed by case-control status and the results are shown in Table 3. The average IHC score per person for TT cases and controls for each molecular marker is also represented in Fig 1. Staining was generally highest in the epithelium of TT cases. CTGF, IL-1β and CC1 had greater average expression in TT cases in both epithelial and subepithelial compartments.

**Table 3:** Expression of specific molecular markers in the epithelial and subepithelial compartments by IHC.

		Epithelial Compartment			Subepithelial Compartment		
Category	Target	Cases (N = 20)	Controls (N = 16)	P*	Cases (N = 20)	Controls (N = 16)	P*
**Pro-inflammatory mediators**	**IL-1β**			0.312			0.012
	0	9	9		11	14	
	1	1	3		1	2	
	2	8	4		7	0	
	3	2	0		1	0	
	**IL-17A**			1			NA
	0	17	14		20	16	
	1	2	1		0	0	
	2	1	1		0	0	
	3	0	0		0	0	
	**CXCL5**			0.241			NA
	0	14	14		20	16	
	1	4	0		0	0	
	2	2	2		0	0	
	3	0	0		0	0	
	**S100A7**			0.009			NA
	0	11	16		20	16	
	1	5	0		0	0	
	2	1	0		0	0	
	3	3	0		0	0	
	**IL-22**			0.280			0.806
	0	9	11		16	14	
	1	9	3		1	1	
	2	2	2		3	1	
	3	0	0		0	0	
	**IL-6**			NA			NA
	0	19	15		20	16	
	1	1	1		0	0	
	2	0	0		0	0	
	3	0	0		0	0	
**Inflammasome**	**CC1**			0.213			0.197
	0	9	12		11	14	
	1	4	3		5	1	
	2	4	1		2	1	
	3	3	0		2	0	
**Growth Factors**	**PDGF**			0.689			0.541
	0	12	7		15	12	
	1	4	4		3	3	
	2	4	4		2	0	
	3	0	1		0	1	
	**TGFβ2**			0.359			0.037
	0	13	7		19	12	
	1	1	4		0	4	
	2	4	4		0	0	
	3	2	1		1	0	
	**CTGF**			0.008			0.099
	0	2	10		5	7	
	1	6	2		8	5	
	2	11	3		2	4	
	3	1	1		5	0	
**Matrix**	**MMP9**			0.263			0.556
	0	11	8		15	10	
	1	3	0		2	1	
	2	6	7		3	5	
	3	0	1		0	0	
	**MMP7**			NA			NA
	0	18	16		20	14	
	1	2	0		0	2	
	2	0	0		0	0	
	3	0	0		0	0	
**EMT**	**E-Cadherin**			0.314			
	0	1	0				
	1	0	3				
	2	2	2				
	3	3	1				
	4	14	10				
	**Vimentin**			0.180			
	**0**	0	3				
	1	7	5				
	2	8	7				
	3	5	1				
	**αSMA**			1			
	0	12	11				
	1	1	0				
	2	5	4				
	3	2	1				

* Fishers exact test was used to test for differences between groups. Unadjusted P values are shown. To account for the burden of repeated statistical tests applied, critical p-value thresholds of (0.05/13) <0.004 for the epithelial compartment and (0.05/7) <0.007 in the subepithelial compartment would be required.

**Fig 2:**
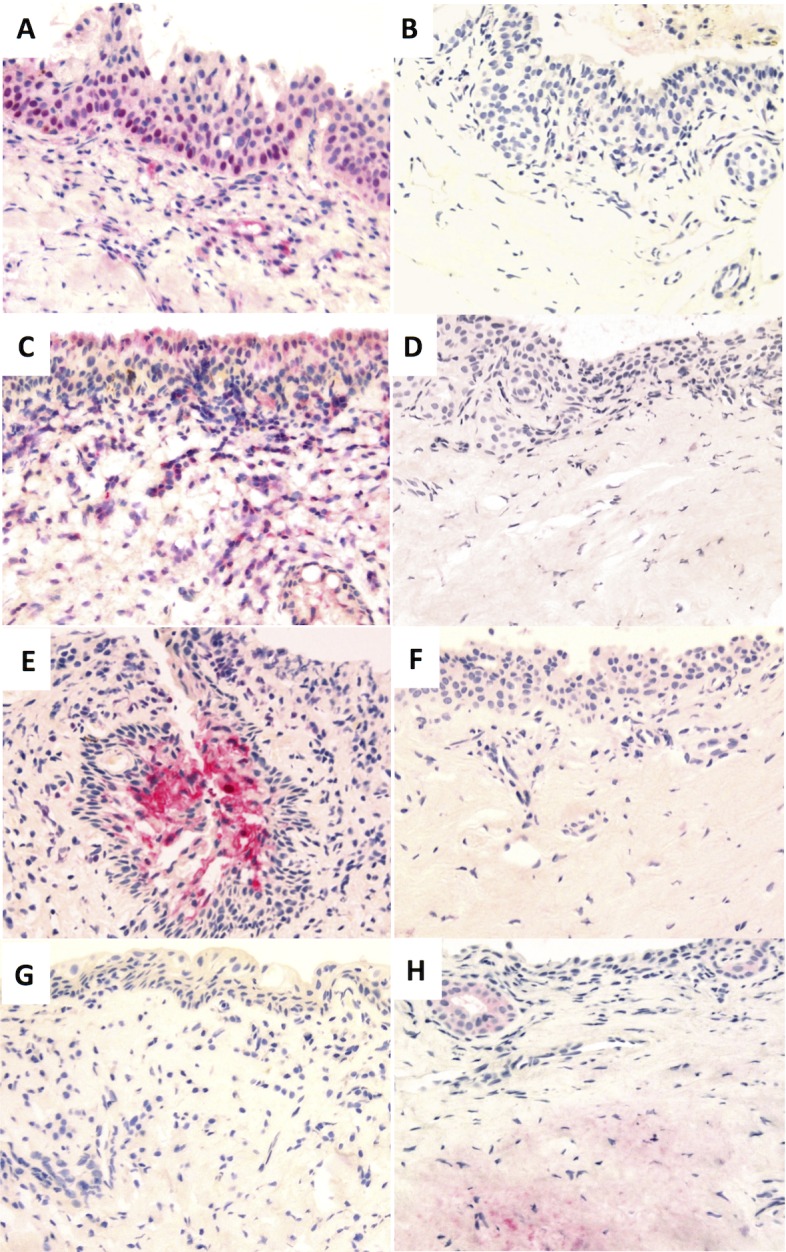
Example images of IHC staining. CTGF in the deep epithelium of TT cases (A) and controls (B), IL-1β in TT cases (C) and controls (D), S100A7 in a pseudogland of Henle of a TT case (E) and S100A7 staining in controls (F) and TGFβ2 staining in TT cases (G) and in controls (H). Images were taken at 200x original magnification.

CTGF expression was greater in the epithelium of TT cases relative to controls (P = 0.0085). Of the samples that scored >0 for CTGF, 10/23 had a clinical papillary inflammation grade >0. Epithelial expression of CTGF was localized in 14/24 of the samples that stained positive. In 4/24 CTGF positive samples (two cases, two controls) expression was more concentrated in the deep epithelium (Fig 2A). CTGF expression was slightly greater in the subepithelial tissue of cases but the difference was not significant.

Significantly more expression of IL-1β was detected in the subepithelial tissue of TT cases relative to controls (P = 0.012). IL-1β expression was localized around the inflammatory cell infiltrate (Fig 2C), however, only 5/11 of samples that stained >0 for IL-1β had a clinical papillary grade >0. Expression of IL-1β tended to be higher in the epithelium of TT cases but the difference was not statistically significant.

S100A7 expression was significantly higher in the epithelium of TT cases (P = 0.0095). Expression of S100A7 within the epithelium was patchy and in 2 samples expression was localized to pseudoglands of Henle (Fig 2E). All controls stained negative for S100A7. Six of the nine samples that scored >0 for S100A7 had a clinical papillary grade >0. S100A7 was not detected in the subepithelial tissue in any sample.

TGFβ2 expression was slightly greater in the subepithelial tissue of controls (P = 0.037). Four controls were weakly positive (Fig 2H) and one case (papillary grade 3) had stronger expression of TGFβ2 in the subepithelial tissue. For the remaining 31 samples TGFβ2 was not detected in the subepithelial tissue. Epithelial expression of TGFβ2 was not different between cases and controls.

There were no statistical differences in the expression of EMT biomarkers E-cadherin, vimentin and αSMA in the epithelium between cases and controls. There were also no statistical differences between cases and controls in epithelial or subepithelial tissue expression of CC1, MMP9, PDGF, IL-17A, IL-22 and CXCL5 (Table 3). IL-17A and CXCL5 were not detected in the subepithelial tissue in any samples. IL-6 and MMP7 were detected in ≤2 of the 36 samples tested.

## Discussion

In this study we found that conjunctival tissue of TT cases had significantly greater S100A7 and CTGF expression in the epithelium and significantly greater IL-1β expression in the subepithelial tissue. The average expression of CTGF, IL-1β and CC1 was greater in TT cases in both epithelial and subepithelial compartments. Controls tended to have more expression of TGFβ2 in the subepithelial tissue. We did not detect an increase in the expression of EMT biomarkers in the epithelium of samples from individuals with TT. We found that individuals with TT had different patterns of collagen deposition and an increased inflammatory cell infiltrate in the subepithelial compartment relative to individuals without clinical evidence of trachoma.

In this study the age distribution of cases and controls was comparable. There were more females among the TT cases, however, this was not a statistically significant. There were substantially more Maasai people among the cases. This probably reflects greater environmental and behavioral risk factors [20,21]. Maasai people live in close contact with their livestock (flies are often abundant) and in areas with fairly limited access to water. Furthermore the uptake of antibiotics for trachoma control may be lower in these communities [22]. Although genetic risk factors in Maasai people cannot be excluded, the behavioral and environmental risk factors leading to increased exposure to *C*. *trachomatis* infection probably account for the higher proportion of Maasai among TT cases.

Changes in tissue morphology were clearly evident with a transition from a “block” type pattern of collagen deposition in controls to “wavy” and “fine” type patterns in cases. This probably reflects the progressive disruption of normal connective tissue. Degradation of organized bundles of collagen fibers running parallel with the epithelium (“block” type) by MMPs or oedema could create the fragmented “wavy” and “fine” patterns observed. A similar observation has previously been shown in the subepithelial tissue of individuals with scarring trachoma and was found to correlate with tissue scarring determined by *in vivo* confocal microscopy (IVCM) [23]. There is an apparent mismatch in the features of TS when tissue is observed by the 2 different methods; using IVCM, defined bands of scarring were observed, whereas by histology collagen bundles appear fragmented and amorphous. It is possible that the bands of scarring (observed by IVCM in 3 dimensions) disrupt the parallel collagen bundles (seen on a section in 2 dimensions) to produce wavy and fine patterns of collagen in cases. It was not possible to grade fibrosis in subepithelial tissue with the same grading system described in Hu *et al* [23] as tissue sections were not sufficient in size. The baseline “T” collagen structure indicative of healthy tissue in controls was visible only in one control section.

More inflammatory cells were identified in tissue from TT cases. This result is in keeping with the range of clinical inflammatory grades observed in cases, whereas all controls had a clinical inflammation grade of 0. It is perhaps surprising that epithelial atrophy, thought to be a common feature of scarring trachoma, and the number of myofibroblasts were not different between cases and controls. Epithelial atrophy has been reported in two studies that used samples from 11 and 29 individuals with TT and entropion, however neither study included controls [24,25]. The same two studies also reported epithelial hyperplasia and psuedogland formation [24,25]. Persistent and recurrent conjunctival inflammation and associated hyperplasia are thought to promote the formation of pseudoglands of Henle, which are crypts formed by invagination of the epithelium [26]. Bacteria and debris can become trapped by mucus within these crypts and entrapped secretions within pseudoglands were observed in individuals with TT [24,26]. Myofibroblasts have contractile properties therefore one might speculate that they have an increased role during TS and TT. In line with our observations we did not detect any significant differences in the epithelial expression of the EMT biomarkers vimentin, αSMA or E-cadherin between cases and controls, although vimentin expression was slightly increased in TT cases. The number of samples in this study was relatively small and there was only one sample from an individual with *trachomatous inflammation–intense*, therefore it is possible we did not have sufficient power within the study to detect subtle, transient or rare events. We only graded loss of E-cadherin and gain of vimentin and αSMA expression in the epithelium, as it would not be possible to distinguish cells expressing vimentin and αSMA in the subepithelial tissue from normal fibroblasts/myofibroblasts. Future work such as multiplex staining or application of new techniques such as laser ablation mass cytometry are required to distinguish complex cell phenotypes and rare events such as cells undergoing EMT [27].

IHC staining was generally greater in the epithelial compartment relative to the subepithelial compartment. CC1, CTGF and IL-1β were increased in both epithelial and subepithelial compartments in TT cases (Fig 1) and S100A7 was increased in the epithelium. CC1 cleaves IL-1β into its active form and the concomitant upregulation of CC1 and IL-1β reflects activation of the inflammasome [28]. In the subepithelial tissue of TT cases IL-1β was localized around the inflammatory cell infiltrate. Just over half of the samples that stained positive for IL-1β in the subepithelial tissue had no evidence of clinical inflammation; therefore considerable levels of IL-1β were expressed in the absence of clinical signs. Recent evidence showed that IL-1β expression was weakly associated with progressive scarring trachoma and strongly associated with inflammatory episodes [1]. It is possible that IL-1β remains up-regulated in the subepithelial tissue in individuals without evidence of clinical inflammation, as this study might suggest, but that cytokines expressed in the subepithelial tissue are less readily detected when samples are collected using a superficial conjunctival swab. Chronic IL-1 induced inflammation is known to result in tissue remodeling [29,30].

CTGF modulates the interaction of cells with the extracellular matrix; promoting collagen deposition, mesenchymal cell activation and differentiation (including EMT) and tissue remodeling [14,31]. CTGF was previously shown by IHC to be upregulated in infiltrating monocytes/macrophages of children with active trachoma [12], however we demonstrate an upregulation of CTGF in both the subepithelium and epithelium of TT cases. This difference could reflect the different clinical stages of trachoma in the samples studied. TGFβ induces CTGF expression in fibroblasts and epithelial cells therefore it is surprising that we did not see a concomitant up-regulation of TGFβ in TT cases alongside CTGF [32–34]. A number of bacteria have been shown to stimulate CTGF expression in epithelial cells via the lysophosphatidic acid receptor [35], therefore it is possible that CTGF is directly induced in the epithelium by the altered ocular microbiota observed in individuals with trachoma [18,36]. Over-expression of CTGF drives fibrosis in a number of diseases [32,37,38] and it has become apparent that epithelial-derived CTGF can drive fibrosis in the underlying subepithelial tissue [34,39]. CTGF was detected in the basal epithelium in four samples (Fig 2A). CTGF staining in the basal epithelium has previously been reported in the context of gingival fibrosis, where it was thought to have a role in cell proliferation and epithelial hyperplasia [40]. This could drive the formation of pseudoglands in addition to driving fibrosis in the underlying tissue. CTGF was strongly associated with clinical inflammation in adults with progressive scarring trachoma [1], however in the present study CTGF did not appear to be preferentially detected in adults with evidence of clinical inflammation.

S100A7 is a pro-inflammatory antimicrobial peptide secreted by epithelial cells. S100A7 was only detected in the epithelium of TT cases in this study and expression was generally patchy, possibly suggesting a localized antimicrobial response. In addition to direct antibacterial action, S100A7 recruits CD4+ T cells and neutrophils and amplifies pro-inflammatory cytokine responses [41–43]. In two samples staining was detected around pseudoglands of Henle, possibly reflecting a local inflammatory response to bacteria that had accumulated within the pseudogland. Positive staining of the epithelium around these pseudoglands was also noted for IL-1β in 4 TT cases. No positive staining was detected around pseudoglands in control tissue for any of the antibodies tested. Due to the small size of the tissue sections it was not possible to compare the number of pseudoglands between cases and controls. Further study is required to identify whether trachomatous inflammation promotes pseudogland formation and whether bacteria trapped within pseudoglands have a role in exacerbating inflammation.

Latent TGFβ2, PDGF and MMP9 expression tended to be slightly higher in controls. TGFβ2 expression in controls was relatively weak and non-specific therefore could be attributed to a high background. The antibody used detected the latent form of TGFβ2, therefore it is possible that more latent TGFβ2 was present in controls whereas cases had activated and released TGFβ2. Despite having a well-defined role in tissue fibrosis, no previous associations have been found between TGFβ2 and trachoma at both expression and protein levels (Holland, Mabey and Bailey; personal communication) [44]. Full characterization of the role of TGFβ2 in trachoma has been limited due to its complex post-translational modifications.

IL-17A, CXCL5, PDGF, MMP7 and MMP9 have previously been associated with various stages of trachoma (*trachomatous inflammation–follicular* [10,11,44,45], TS [1,6,7] and TT [8,9]) at mRNA and protein expression levels, however they were not demonstrably up-regulated in TT cases in the present study. MMP7, CXCL5 and IL-17A were strongly associated with inflammatory episodes but not with progressive scarring in two large cohorts of individuals with trachoma [1]. The failure to detect differences in staining in this study could be due to the lack of clinical inflammation in the individuals from whom samples were obtained, or due to a lack of study power to detect more subtle differences. It could also be biological and might suggest that differences at the expression level are not maintained at the protein level. The failure to detect MMP7 and IL-6 could likewise be due to a lack of clinical inflammation, a lack of expression or due to the sensitivity of the antibodies used. IL-22 is released alongside IL-17 by Th17 cells and contributes to mucosal defense and maintenance of epithelial integrity but also to the pathogenesis of psoriasis [46,47]. Although IL-22 has not previously been associated with trachoma we hypothesized it might have a role in conjunctival epithelial inflammation or health [48], however no differences in expression were detected.

We did not to collect swabs for *C*. *trachomatis* PCR because the swabbing process would have probably altered the surface tissue appearance. However, from contemporary studies it is known that the prevalence of *C*. *trachomatis* infection in this region among individuals with trachomatous scarring is very low, and therefore it is likely that few if any of these case would have been infected [1]. Similarly, it was not possible to collect conjunctival swab samples for mRNA gene expression analysis from these individuals as this would have affected the histological analysis. Our previous gene expression work used mRNA collected from surface swabs. Therefore, we would not necessarily expect these to exactly correspond to this immunohistochemistry study, which is assessing protein mostly in deeper levels.

**Fig 3:**
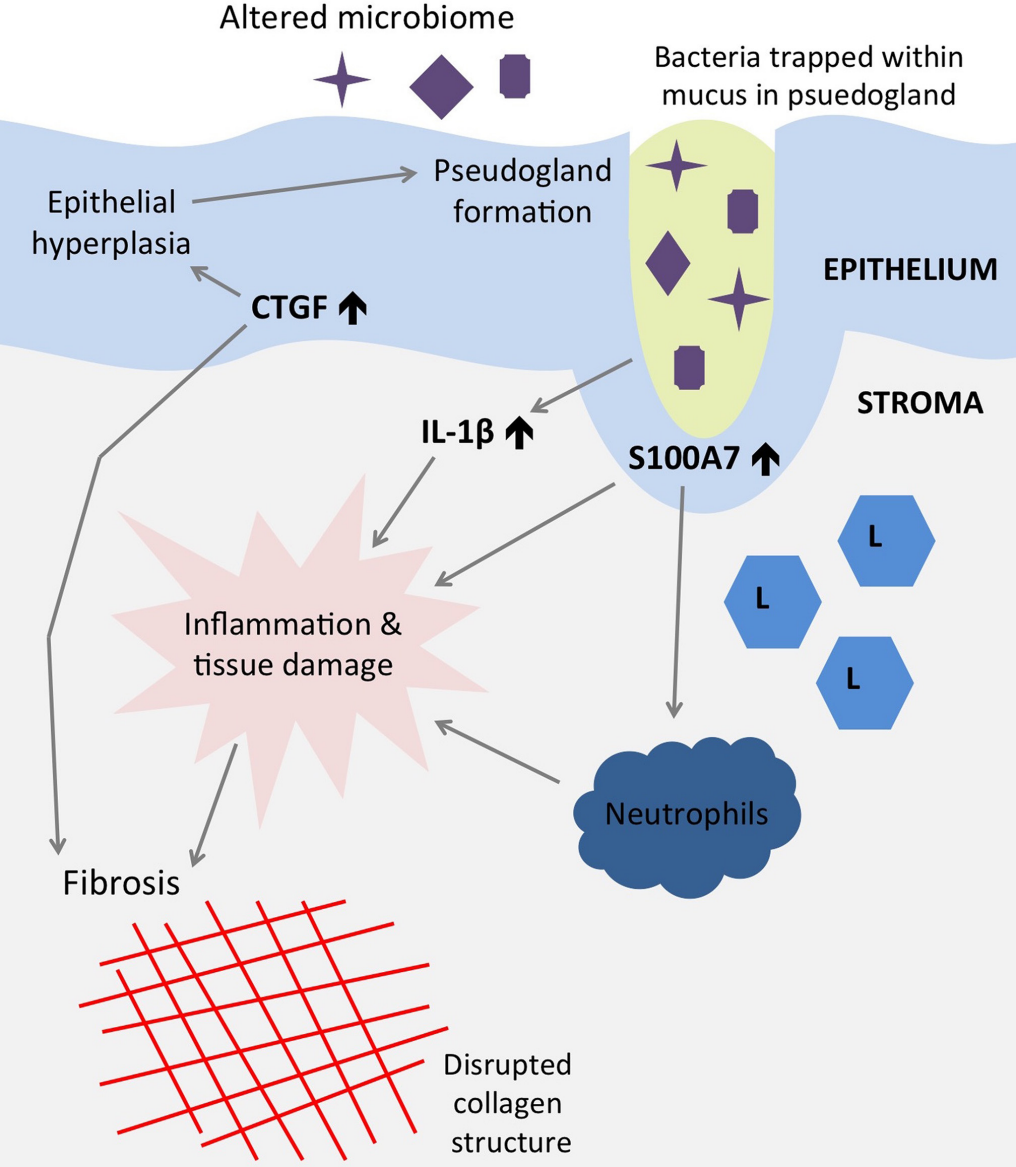
Model figure summarizing molecular marker expression, inflammatory cell infiltration and tissue morphology changes observed in trichiasis tissue, including hypothesized mechanisms driving scarring. “L” = lymphocytes.

We have demonstrated that individuals with TT had significantly increased levels of CTGF and S100A7 in the epithelium and IL-1β in the subepithelial tissue, even in the absence of marked clinical inflammation. CTGF, IL-1β and CC1 were increased in TT cases in both epithelial and subepithelial compartments. We suggest that microbial stimulation of the epithelium, ongoing sub-clinical inflammation and inflammasome activation in the connective tissue and CTGF-driven fibrosis contribute to the pathology of trachoma. We also described a potential role for pseudoglands of Henle in trachoma that warrants further investigation. These results and hypothesized mechanisms are summarized in a model figure (Fig 3). CTGF could be responsible for driving inflammation-induced fibrosis in trachoma, making it a potential therapeutic target [49]. Future research should focus on the stimuli that lead to up-regulation of CTGF, S100A7 and IL-1β and potential inhibitors that could halt the progression of scarring.

## Supporting Information

S1 Table Antibodies and retrieval methods used in this study. (DOCX)

S1 Fig Cross-polarized light images of haemotoxylin and eosin stained tissue sections. Example images representative of “block” (A), “wavy” (B) and “fine” (C) patterns of fibrosis are shown. Images were taken at 200X original magnification.
(TIFF)
