# Supplementary material for: Increased Epithelial Expression of CTGF and S100A7 with Elevated Subepithelial Expression of IL-1β in Trachomatous Trichiasis
Source: PLoS Negl Trop Dis. 2016 Jun 1;10(6):e0004752. doi: 10.1371/journal.pntd.0004752 (PMC4889093; doi:10.1371/journal.pntd.0004752)
Supplement: S1 Table — (DOCX) [file pntd.0004752.s001.docx]

| **Category** | **Target** | **Swiss-Prot ID** | **Dilution** | **Retrieval** | **Supplier** | **Antibody** |
| --- | --- | --- | --- | --- | --- | --- |
| **Pro-inflammatory mediators** | IL-1β | P01584 | 1:75 | H2*(20)^‡^ | ABCAM | AB2105 |
|  | IL-6 | P05231 | 1:400 | NR^§^ | LEICA | NCL-L-IL6 |
|  | IL-17A | Q16552 | 1:350 | H1^†^(30) | ABCAM | AB136668 |
|  | CXCL5 | P42830 | 1:200 | H2(20) | ABCAM | AB9802 |
|  | S100A7 | P31151 | 1:400 | H1(10) | ABCAM | AB13680 |
|  | IL-22 | Q9GZX6 | 1:100 | H2(20) | ABCAM | AB18499 |
|  |  |  |  |  |  |  |
| **Growth Factors** | PDGF | P04085 | 1:200 | H2(20) | ABCAM | AB178409 |
|  | TGFβ2 | P61812 | 1:100 | H1(20) | ABCAM | AB36495 |
|  | CTGF | P29279 | 1:300 | H1(10) | ABCAM | AB6992 |
|  |  |  |  |  |  |  |
| **EMT** | E-cadherin | P12830 | 1:200 | H2(20) | DAKO | NCH-38 |
|  | Vimentin | P08670 | 1:3000 | H2(20) | DAKO | M0725 |
|  | αSMA | P62736 | 1:500 | NR | DAKO | M0851 |
|  |  |  |  |  |  |  |
| **Matrix** | MMP7 | P09237 | 1:50 | H2(20) | ABCAM | AB4044 |
|  | MMP9 | P14780 | 1:200 | H2(20) | LEICA | NCL-MMP9-439 |
|  |  |  |  |  |  |  |
| **Inflammasome** | CC1 | P29466 | 1:50 | H2(20) | ABCAM | AB1872 |

**H2 = Bond (Leica) epitope retrieval solution 2; pH9 EDTA based buffer and surfactant at 25°C.*

^‡^*(10) = 10 minutes; (20) = 20 minutes; (30) = 30 minutes.*

^§^*NR = no retrieval.*

^†^*H1 = Bond (Leica) epitope retrieval solution 1; pH6 citrate based buffer and surfactant at 25°C.*
