# Supplementary figures and images for: Increased Epithelial Expression of CTGF and S100A7 with Elevated Subepithelial Expression of IL-1β in Trachomatous Trichiasis
Source: PLoS Negl Trop Dis. 2016 Jun 1;10(6):e0004752. doi: 10.1371/journal.pntd.0004752 (PMC4889093; doi:10.1371/journal.pntd.0004752)

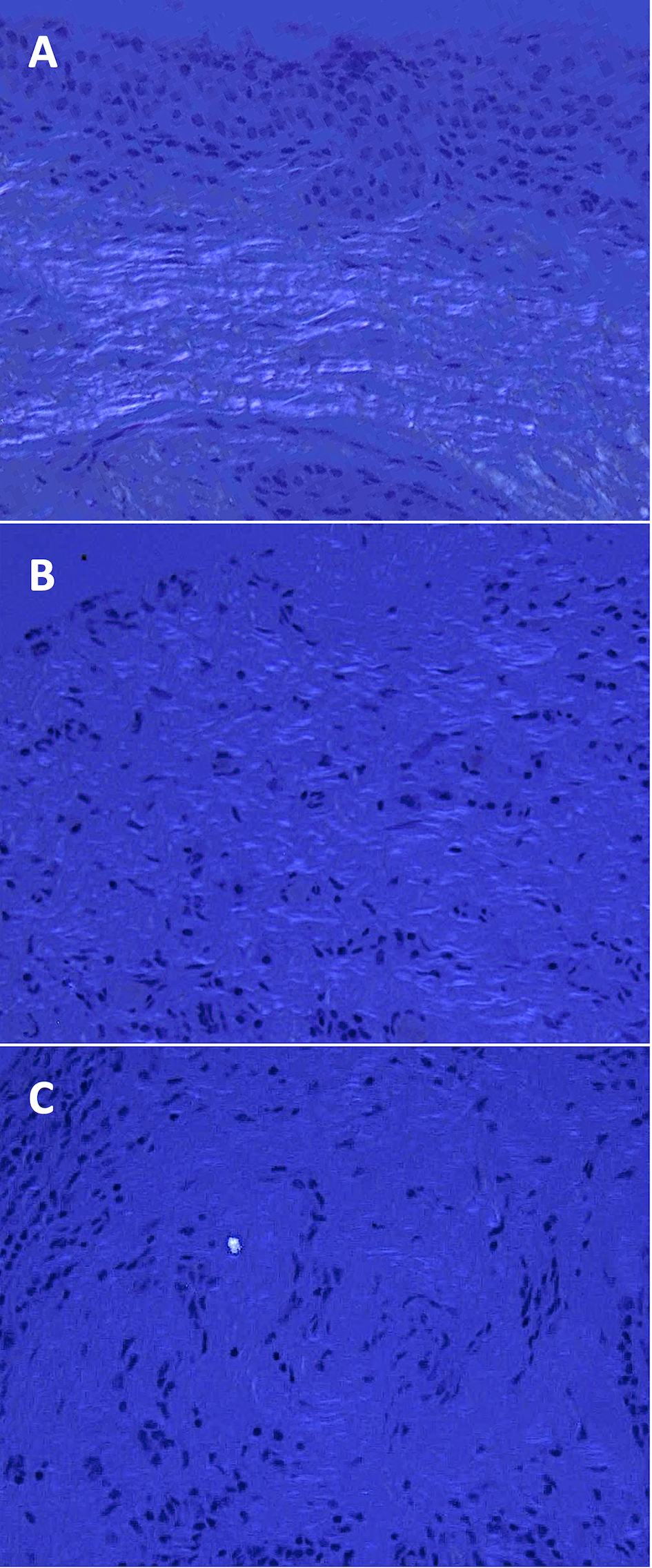

Supplement: S1 Fig — Example images representative of “block” (A), “wavy” (B) and “fine” (C) patterns of fibrosis are shown. Images were taken at 200X original magnification. (TIFF) [file pntd.0004752.s002.tiff]
